# Progressive Extension of a Post-intubation Tracheal Laceration Presenting As Massive Pneumomediastinum: A Case Report

**DOI:** 10.7759/cureus.114478

**Published:** 2026-08-13

**Authors:** Duarte Marques, Maria Pais, Carolina Correia, Hugo Moreira

**Affiliations:** 1 Intensive Care Medicine, Hospital de São Francisco Xavier, Unidade Local de Saúde de Lisboa Ocidental, Lisbon, PRT; 2 Internal Medicine, Hospital Egas Moniz, Unidade Local de Saúde de Lisboa Ocidental, Lisbon, PRT; 3 Internal Medicine, Hospital de São Francisco Xavier, Unidade Local de Saúde de Lisboa Ocidental, Lisbon, PRT

**Keywords:** endotracheal intubation, post-intubation complications, secondary pneumomediastinum, subcutaneous emphysema, tracheal rupture

## Abstract

Post-intubation tracheal laceration is rare and is usually managed conservatively, provided the tear is protected from positive pressure, whether by spontaneous ventilation, by a cuff sited beyond the lesion, or by ventilation delivered without loss of tidal volume. We describe a woman in her early 70s intubated at a district hospital for progressive impairment of consciousness. Imaging before intubation already showed a hiatal hernia, right bronchial mucous plugging, lobar consolidation and bilateral effusions, and initial ventilation delivered 500 mL at a rate of 18 per minute, approximately 9.5 mL/kg of predicted body weight, with an arterial carbon dioxide tension of 28 mmHg. No subcutaneous emphysema was documented before transfer. She arrived at a tertiary unit failing to achieve adequate tidal volumes, with an air leak unresponsive to cuff inflation and extensive cervicothoracic subcutaneous emphysema. The tube was exchanged, and the leak persisted, indicating an airway wall defect. Bronchoscopy showed a longitudinal tear of the posterior membranous trachea, visually estimated at approximately 4 cm and confined to the intrathoracic trachea, with no injury below the tube, and computed tomography confirmed diffuse pneumomediastinum without pneumothorax. The lesion was excluded by cuff position, although the seal varied with head position. Because an unresolved coma made positive-pressure ventilation unavoidable, repair was undertaken. At thoracotomy the following day, the laceration ran from 1 cm above the carina to the thoracic inlet with cervical extension, involving a segment where no injury had been seen the previous day, and the cervical component could not be reached. A persistent leak prompted tube exchange over an airway exchange catheter under simultaneous videolaryngoscopy; the catheter passed extraluminally through the unrepaired tear and the airway was lost. It was regained only after thoracotomy closure and repositioning supine, but desaturation during those attempts had progressed to cardiac arrest, and death was declared intraoperatively. A tracheal laceration may extend between endoscopic assessment and operation, and glottic visualisation does not prevent a false passage when a cervical tear remains unrepaired.

## Introduction

Pneumomediastinum in a critically ill patient should prompt assessment for airway disruption. It may arise from alveolar rupture with air tracking centrally along bronchovascular sheaths, the mechanism described by Macklin [1,2], or from direct disruption of the central airway. Alveolar rupture is recognised in ventilated patients with parenchymal disease and in those generating high transpulmonary pressures through spontaneous effort, in whom pneumomediastinum has been proposed as a surrogate of self-inflicted lung injury in COVID-19 [3,4]. Barotrauma occurred in about one in six patients ventilated for COVID-19 acute respiratory distress syndrome in a systematic review, while pneumomediastinum occurred in about one in nine [5]. Because both mechanisms can produce pneumomediastinum without pneumothorax, the radiological pattern alone does not distinguish them.

Tracheal rupture after endotracheal intubation is uncommon. Reported incidence varies widely, from a historical estimate of about one in 20,000 intubations to between 0.05% and 0.37% of orotracheal intubations in estimates from the decade before 2009 [6]. That literature is dominated by case series in which double-lumen tubes are heavily over-represented relative to general intubation practice, accounting for almost half of the pooled cases [6]. Female sex, age over 65 years, and emergency rather than elective intubation are the most consistently reported risk factors, while long-term inhaled corticosteroid therapy is described as weakening the membranous wall [7]. The posterior membranous trachea, which lacks cartilaginous support, is the usual site [6,7]. Bronchoscopy is the mainstay of diagnosis [7,8].

Practice has moved decisively towards conservative treatment. A morphological classification grades lacerations by depth and by the presence of mediastinal or oesophageal soft-tissue herniation, oesophageal injury, or mediastinitis, and in the single-centre series that applied its revised form, non-operative management was used for the great majority [9,10]. That approach depends on protecting the tear from positive pressure while it heals, whether by spontaneous ventilation, by a cuff sited beyond the lesion, or by ventilation delivered without loss of tidal volume.

We describe a laceration seen at bedside bronchoscopy and estimated at approximately 4 cm, confined to the intrathoracic trachea, which at operation the following day extended from 1 cm above the carina to the thoracic inlet with cervical involvement. The case illustrates that the extent seen at first assessment may not predict the extent at operation, that pneumomediastinum without pneumothorax is not specific for airway injury, and that when positive-pressure ventilation cannot be discontinued, the precondition for conservative management is absent.

## Case presentation

A previously independent woman in her early 70s presented to a district hospital emergency department with progressive confusion. Her history comprised atrial fibrillation, chronic angina, hypertension, and hypertensive heart disease, without a prior diagnosis of ischaemic cardiomyopathy, and her regular medication included inhaled budesonide, formoterol and glycopyrronium, apixaban, bisoprolol, isosorbide dinitrate, and atorvastatin. Four days earlier, she had attended the same department with a urinary tract infection and decompensated heart failure and had been discharged on cefuroxime. She was found fallen at home twice in the interval, alert and orientated on the first occasion and confused and incontinent of urine on the second, which prompted the index presentation. No head injury was documented on either occasion.

She was disorientated but had no respiratory distress in room air. Blood pressure was 98/48 mmHg with a mean arterial pressure of 65 mmHg, capillary refill time was 3 to 4 seconds, and there was jugular venous distension with hepatojugular reflux, pitting oedema of both legs, and breath sounds reduced over the lower two-thirds of the right hemithorax with scattered wheeze on the left. Laboratory and arterial blood gas findings are shown in Table 1. The room air arterial oxygen tension (PaO_2_) of 148 mmHg was queried in the chart at the time and exceeds the maximum alveolar oxygen tension at that arterial carbon dioxide tension (PaCO_2_), about 106 mmHg at sea level. A respiratory virus panel was negative.

**Table 1:** Laboratory and arterial blood gas findings All values were obtained at presentation to the referring hospital, except the peak lactate, which is the maximum recorded during the intensive care admission.

Finding	Result	Adult reference range
Haemoglobin	9.4 g/dL	12.0-15.0 g/dL
MCV (mean corpuscular volume)	79 fL	80.0-96.1 fL
Leucocytes	4.8 × 10⁹/L	4.0-10.0 × 10⁹/L
Lymphocytes	0.3 × 10⁹/L, 6% of leucocytes	20.0%-40.0% of leucocytes
Platelets	80 × 10⁹/L	150-400 × 10⁹/L
AST (aspartate aminotransferase)	87 U/L	< 32 U/L
ALT (alanine aminotransferase)	67 U/L	< 33 U/L
CRP (C-reactive protein)	6.7 mg/L	< 5.0 mg/L
Urea	96 mg/dL	17-49 mg/dL
Creatinine	0.97 mg/dL	0.50-0.90 mg/dL
Creatine kinase	409 U/L	< 170 U/L
pH in room air	7.43	7.350-7.450
PaCO_2_ (arterial carbon dioxide tension) in room air	35 mmHg	35.0-45.0 mmHg
PaO_2_ (arterial oxygen tension) in room air	148 mmHg	75.0-100.0 mmHg
Lactate	1.4 mmol/L	0.50-2.00 mmol/L
Peak lactate during intensive care admission	2.1 mmol/L	0.50-2.00 mmol/L

Brain computed tomography showed no acute lesion, with incipient ischaemic leukoencephalopathy, and computed tomographic angiography showed normal intracranial vessels and no venous thrombosis. Thoracic computed tomography, obtained before intubation, showed cardiomegaly with a pericardial effusion up to 1.5 cm, a large hiatal hernia containing the gastric body and fundus, epiploic fat, and the tail of the pancreas, a mucoid plug obliterating the lumen of the right bronchial tree, consolidation of the right middle lobe, bilateral pleural effusions of up to 3 cm on the right and 2.5 cm on the left with compressive atelectasis, marked dorsal kyphoscoliosis, and diffuse idiopathic skeletal hyperostosis.

Over the following two days, consciousness fell from a Glasgow Coma Scale score of 14 to 7, with right upper limb hypertonia and bilateral myoclonus. Non-convulsive status epilepticus was the working diagnosis. Antibiotics were stopped after two days, and levetiracetam and lacosamide were started. Lumbar puncture was not performed in the absence of meningism. Consciousness deteriorated further to a score of 6.

**Figure 1:**
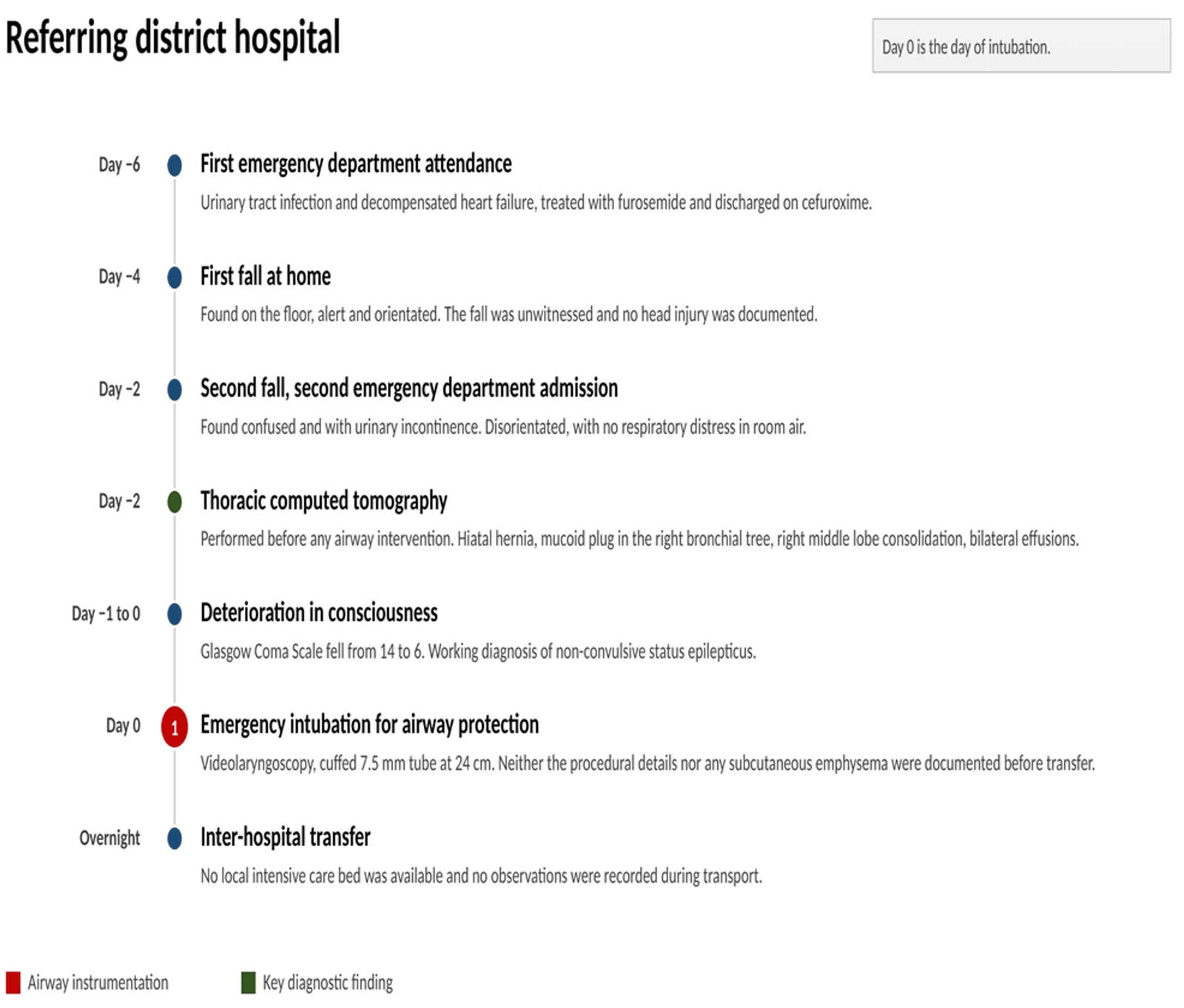
Referring district hospital timeline

Emergency rapid sequence induction was performed for airway protection using videolaryngoscopy, with propofol 50 mg, ketamine 80 mg, and rocuronium 100 mg, and a cuffed 7.5 mm tube was placed at 24 cm at the lips. The videolaryngoscope model and blade, the laryngoscopic view, the use of an introducer, the number of attempts, the method used to confirm tube placement, the seniority of the operator, and any cuff pressure measured at this point were not recorded. What is reported above reached the receiving team as a transcription in the transfer documentation rather than as a primary procedural record. Ventilation was volume-controlled at 500 mL, approximately 9.5 mL/kg of predicted body weight, at a rate of 18 per minute with a positive end-expiratory pressure (PEEP) of 5 cmH_2_O and a fraction of inspired oxygen (FiO_2_) of 0.5, giving pH 7.50, PaCO_2_ 28 mmHg and PaO_2_ 201 mmHg. Noradrenaline was required to 0.74 µg/kg/min, or 66 µg/min at a body weight of 89 kg, and atropine was given for bradycardia at 30 beats per minute attributed to beta-blocker toxicity. No subcutaneous emphysema was documented at any point before transfer. In the absence of a local intensive care bed, she was transferred overnight to a tertiary centre, with no observations recorded during transport (Figure 1).

On arrival, core temperature was 33.8°C and external warming was started. Ventilation was volume-controlled at 420 mL, approximately 8.0 mL/kg of predicted body weight, at a rate of 18 per minute with PEEP 8 cmH_2_O and FiO_2_ 0.30, giving pH 7.37, PaCO_2_ 37 mmHg and PaO_2_ 166 mmHg, with peripheral oxygen saturation 99% and lactate 1.2 mmol/L. She was sedated to a Richmond Agitation-Sedation Scale score [11] of -5 with a bispectral index (BIS, Medtronic, Dublin, Ireland) of 20 and made no triggered breaths. She was desaturating intermittently and failing to achieve adequate tidal volumes, with an audible air leak that did not improve with substantial increases in cuff pressure, extensive cervicothoracic subcutaneous emphysema, and anterior chest wall deformity. The tracheal tube was therefore exchanged under videolaryngoscopic control for a second 7.5 mm tube at 22 cm. No rupture was found in the cuff of the tube removed and the leak persisted, varying with the position of the head. A leak persisting after exchange for a new tube indicated a defect in the airway wall and prompted bronchoscopy.

Flexible bronchoscopy at the bedside, performed before any imaging of the injury, showed a longitudinal tear of the posterior membranous trachea estimated at approximately 4 cm and confined to the intrathoracic trachea, with no tracheal or bronchial injury below the level of the tube. Thick haemopurulent secretions were aspirated, from which influenza A was subsequently isolated. No herniation of mediastinal or oesophageal soft tissue into the tracheal lumen, no loss of substance and no fracture of the tracheal cartilages was described. The extent was a visual estimate made through a trachea occupied by a tube, no endoscopic images were retained, and the estimate is documented in the thoracic surgery consultation note written the same morning.

**Figure 2:**
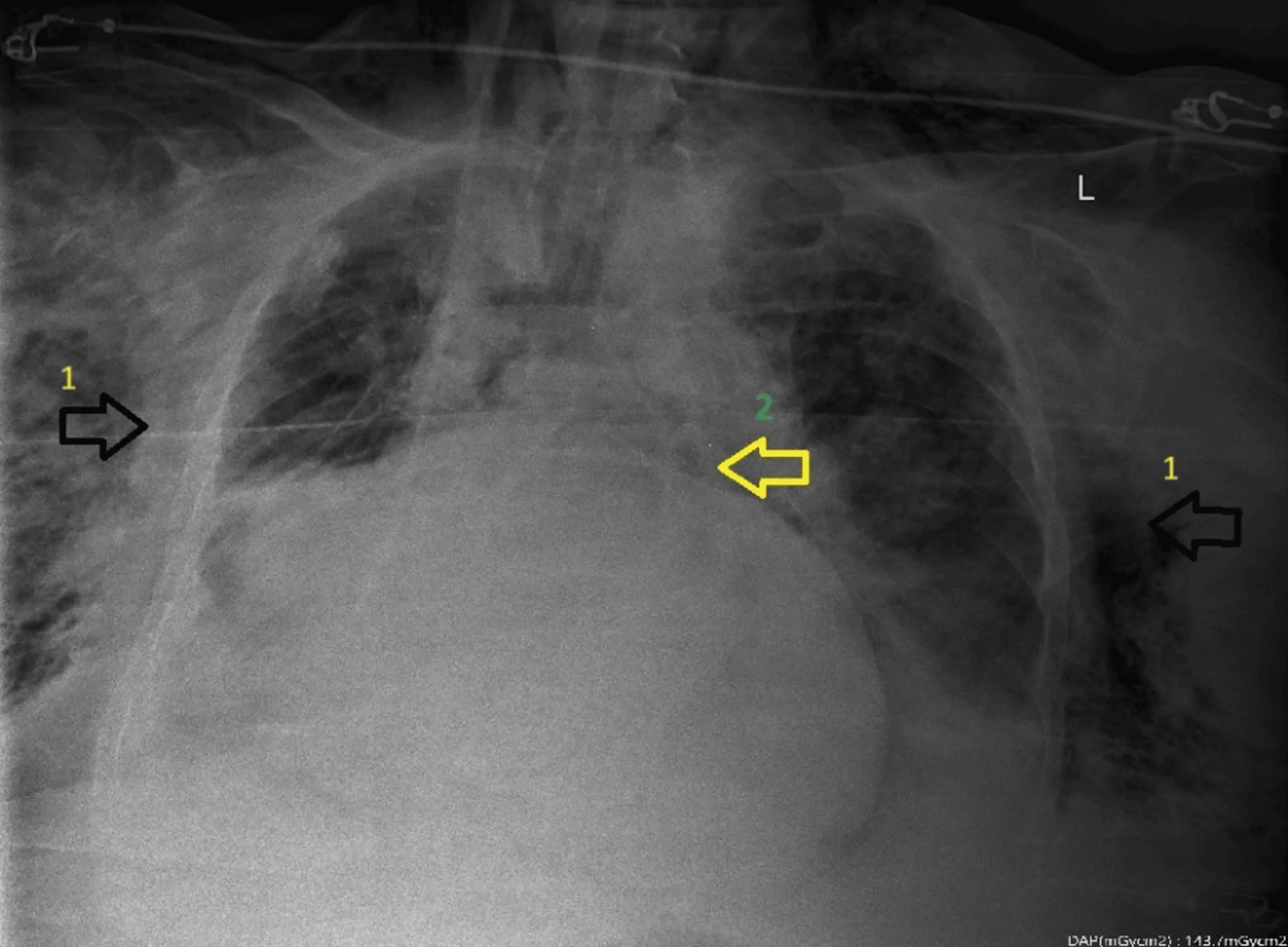
Supine anteroposterior chest radiograph Subcutaneous emphysema (arrow 1, bilateral) and pneumomediastinum (arrow 2).

**Figure 3:**
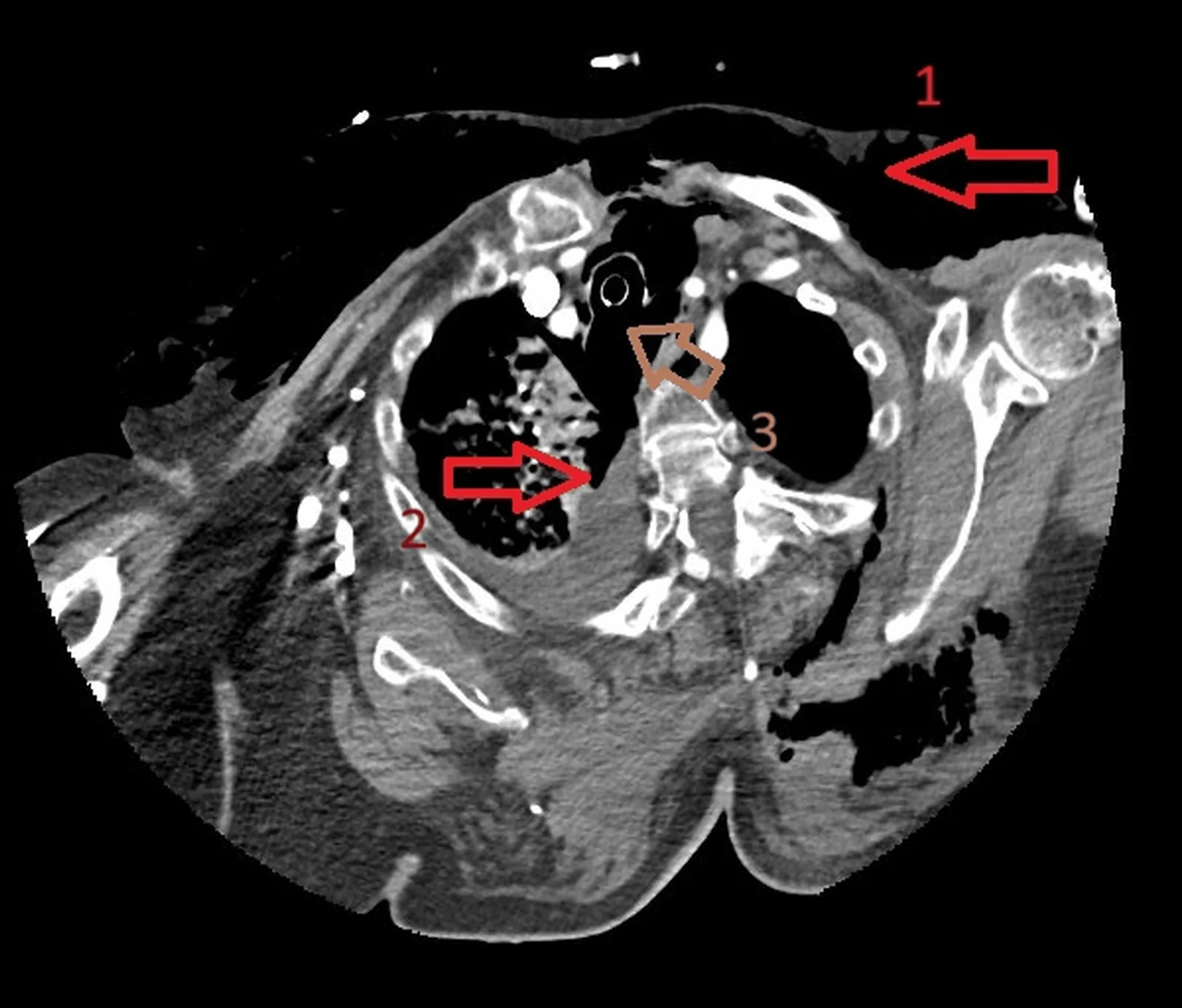
Contrast-enhanced thoracic computed tomography, axial section at the level of the thoracic inlet Subcutaneous emphysema of the anterior chest wall (arrow 1), diffuse pneumomediastinum (arrow 2), and disruption of the posterior membranous tracheal wall (arrow 3).

**Figure 4:**
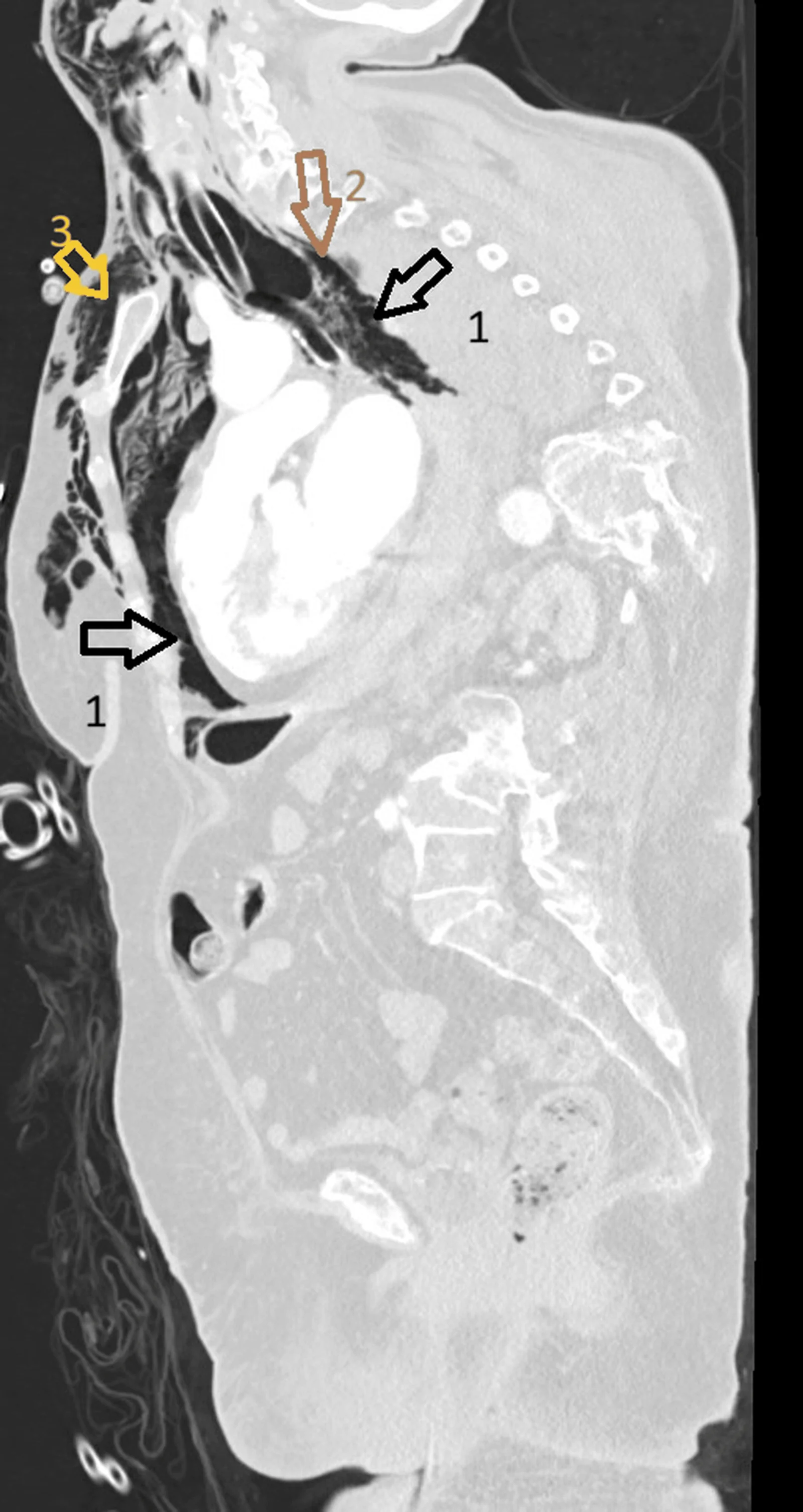
Contrast-enhanced thoracic computed tomography, sagittal reformat Diffuse pneumomediastinum (arrow 1), discontinuity of the posterior membranous tracheal wall (arrow 2) and subcutaneous emphysema of the anterior cervicothoracic soft tissues (arrow 3). The discontinuity corresponds to the lesion identified at bronchoscopy; the full extent found at thoracotomy was not appreciable on this imaging.

Chest radiography raised the possibility of pneumomediastinum (Figure 2). Contrast computed tomography confirmed diffuse pneumomediastinum, extensive subcutaneous emphysema of the thoracic and cervical wall with anterior predominance and extension into both breasts, and discontinuity of the posterior tracheal wall, with the distal end of the tracheal tube lying within the tracheal lumen, no pneumothorax, total collapse of the right middle and lower lobes and moderate bilateral effusions (Figures 3, 4). The leak then ceased with the tube at 22 cm and a cuff pressure of 20 cmH_2_O, which the intensivist recorded at the time as indicating that any tracheal defect lay proximal to the cuff. The tube was left at that depth to exclude the lesion from the ventilating circuit. Upper gastrointestinal endoscopy, requested by the thoracic surgery team later that morning, excluded oesophageal perforation, and a nasogastric tube was placed under direct vision.

**Figure 5:**
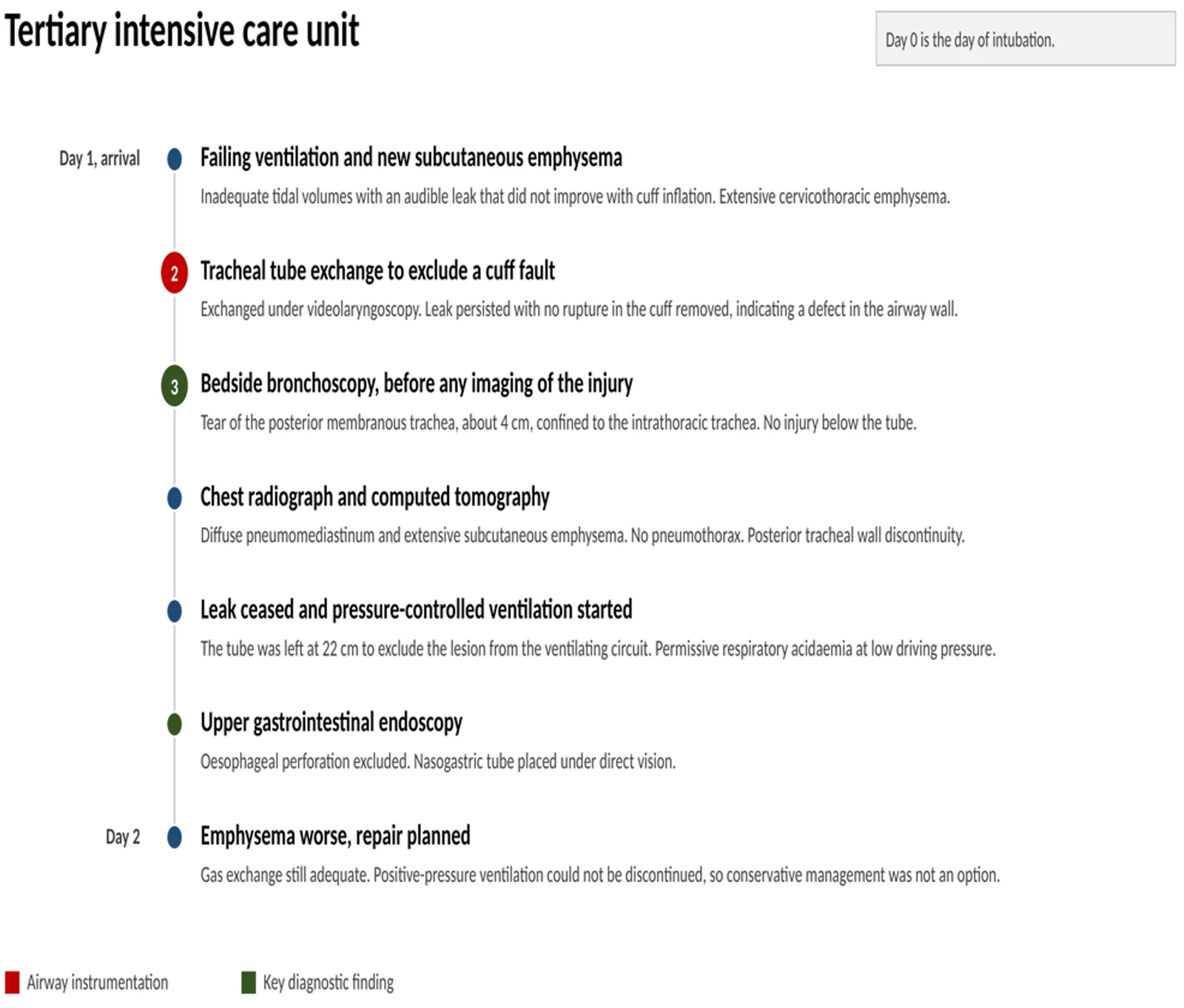
Tertiary intensive care unit timeline

Ventilation was changed to pressure control at 16 cmH_2_O above a PEEP of 6 cmH_2_O, delivering exhaled volumes of about 300 mL, and gases at that setting moved from pH 7.32 with PaCO_2_ 43 mmHg to pH 7.28 with PaCO_2_ 57 mmHg over the following hours, accepted as permissive respiratory acidaemia. By the evening, at 20 cmH_2_O above PEEP 5, exhaled volumes were 350 mL with pH 7.45 and PaCO_2_ 36 mmHg. Electroencephalography, performed with sedation suspended, showed no epileptiform activity. The Glasgow Coma Scale score off sedation was 5, with flexion to pain. Toxicology, including a tricyclic antidepressant screen, was negative. Noradrenaline requirement ranged from 0.11 to 0.45 µg/kg/min through the intensive care admission and peak lactate was 2.1 mmol/L, with no clinical signs of hypoperfusion. After multidisciplinary discussion, repair was planned for the following day, on the grounds of a tracheal perforation maintaining a diffuse pneumomediastinum in a patient who could not be liberated from positive-pressure ventilation. At the time of that decision, the subcutaneous emphysema was recorded as clinically stable and not tense, with no audible leak from the tube, and the marked kyphosis and short neck were confirmed on direct examination (Figure 5).

On the second morning, the emphysema was worse clinically and radiologically but without haemodynamic or ventilatory repercussion. Chest radiography showed markedly worse subcutaneous emphysema and right lung atelectasis attributed to the hiatal hernia. Pressure control at 18 cmH_2_O above PEEP 6 delivered 390 mL, approximately 7.4 mL/kg, with no detectable leak and gases of pH 7.39, PaCO_2_ 42 mmHg and PaO_2_ 105 mmHg at FiO_2_ 0.25, although within the same period, the driving pressure was reduced to 16 cmH_2_O and the inspired oxygen fraction was raised to 0.45. Platelets were 78 to 81 × 10⁹/L with an international normalised ratio of 1.5 and one pool was given, and creatinine rose from 0.97 to 1.64 mg/dL, which we graded retrospectively as stage 1 acute kidney injury, although the discharge summary recorded none. She had completed two days of levofloxacin and cefuroxime before transfer and received no antibiotic in the intensive care unit, where she was deliberately left in an antibiotic window. An isolated febrile spike occurred on the first intensive care day, with C-reactive protein rising from 6.7 to 21.5 mg/L and leucocytes from 4.8 to 12.5 × 10⁹/L. Influenza A, isolated from the bronchial secretions sampled at bronchoscopy despite the negative respiratory virus panel at the referring hospital, was the only positive microbiological result, with blood cultures, urinary antigens, tracheal aspirate cultures, and surveillance screens all negative. Mediastinitis was neither diagnosed clinically nor reported on imaging at any point.

Ventilation deteriorated during transfer to theatre, where adequate ventilation could not be maintained on the transport ventilator, and a frank leak with low delivered volumes was present on arrival in the operating room, while oxygen saturation was still adequate. The documented plan was exchange of the tracheal tube for a laryngeal tube, right axillary thoracotomy through the fourth intercostal space, and intrathoracic guidance of the tube into the left main bronchus to exclude the right lung. The exchange was not performed. Left endobronchial advancement of the existing tube was attempted and could not be achieved. Severe refractory desaturation followed, so two-lung tracheal ventilation was maintained and emergency thoracotomy proceeded in the left lateral decubitus position. The trachea was controlled with proximal reference at the thoracic inlet and distal reference at the carina. The laceration was considerably more extensive than described at bronchoscopy and on computed tomography, running from 1 cm above the carina to the thoracic inlet, with extension into the cervical trachea, and was full thickness. It was closed primarily with a continuous 3/0 polydioxanone suture from the thoracic inlet downwards and buttressed with an autologous pericardial flap. The operative record describes no loss of substance, no fracture of the tracheal cartilages, and no oesophageal involvement. The cervical component could not be reached through the thoracic incision and was left unrepaired; a cervical approach had been judged complex before operation because of marked kyphosis and a short neck.

Intervals of one-lung ventilation needed for exposure were achieved by the surgeon guiding the tube intrathoracically and were very poorly tolerated, with prolonged hypoxaemia and inconsistent recovery even on resuming tracheal ventilation. Electrical instability increased, with atrial fibrillation and a rapid ventricular response and a transient run of ventricular tachycardia, and noradrenaline reached 3.2 mg/h, approximately 0.60 µg/kg/min. At the end of the repair, a leak persisted whose origin in the unrepaired cervical trachea could not be distinguished from rupture of the tube cuff. Exchange for an armoured tube was attempted over an airway exchange catheter under simultaneous videolaryngoscopy, which showed marked supraglottic oedema and a difficult but obtained view. The catheter was nonetheless found to have passed extraluminally through the unrepaired laceration and the airway was lost. Intubation over the catheter was not possible. The thoracotomy was closed as an emergency, and the patient was repositioned supine, after which orotracheal intubation was achieved by videolaryngoscopy after multiple attempts, difficult but successful and secured only at the final stage. During those manoeuvres, desaturation was profound and progressed to cardiac arrest.

**Figure 6:**
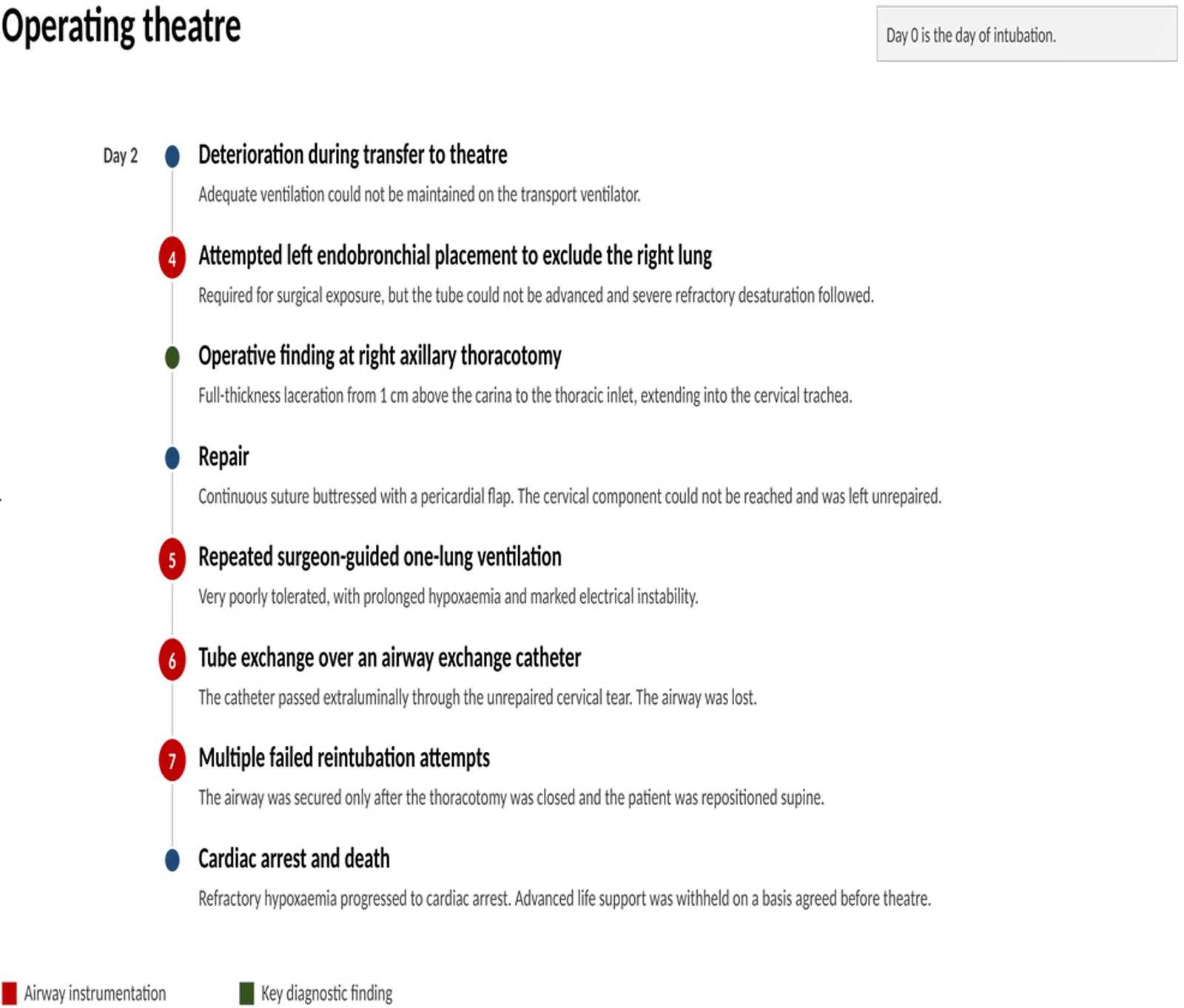
Operating theatre timeline

Advanced life support was withheld rather than withdrawn, on a basis agreed with the intensive care team before theatre and applied at the arrest: an unexplained coma unresolved after two days of investigation and requiring continued ventilation, an unrepaired cervical laceration unreachable through the thoracic approach, repeated severe intraoperative hypoxaemia with marked electrical instability, and a cervical approach judged complex because of the patient's habitus. Death was declared intraoperatively, in the afternoon of the second intensive care day. The exact interval from intubation cannot be given because the time of intubation was not recorded. The family was informed by the surgical team, no post-mortem examination was performed, and the case was reviewed at the institutional morbidity and mortality meeting (Figure 6).

## Discussion

Alveolar rupture with central air tracking cannot be formally excluded. She had parenchymal disease before intubation, and initial ventilation delivered 9.5 mL/kg of predicted body weight, above the range recommended in acute respiratory distress syndrome [12], which she did not have and outside which the advantage of low volumes is not established [13]. The resulting PaCO_2_ of 28 mmHg shows that minute ventilation exceeded requirement. Self-inflicted lung injury, the first of the two mechanisms proposed for pneumomediastinum here, requires spontaneous inspiratory effort [3], and she made none, sedated to a Richmond Agitation-Sedation Scale [11] score of -5 without triggered breaths. Ventilator-delivered barotrauma needs no such effort [5], but the pressures that would bear on it were never recorded at the referring hospital, where ventilation was volume-controlled with no peak or plateau charted. Our own peak pressures, which in pressure control equalled the set inspiratory pressure above PEEP and ranged from 22 to 25 cmH_2_O, were applied to a trachea already torn. A visualised full-thickness laceration accounts for the pneumomediastinum; an alveolar contribution can neither be shown nor excluded. Oesophageal perforation gives a comparable pattern, and contrast-enhanced computed tomography is the examination of choice, with endoscopy reserved for doubtful findings because it risks enlarging the perforation [14]; here, computed tomography could not exclude oesophageal injury against diffuse pneumomediastinum, so endoscopy was performed on that indication and excluded it.

Neither difficult intubation, repeated attempts nor cuff overinflation was documented, although the absence does not exclude significant injury. The most important modifiable risk factors are procedural, while female sex, age over 65 years, and emergency intubation are the most important non-modifiable ones [6,7]. All three were present, as was long-term inhaled corticosteroid therapy, which is described as weakening the membranous wall [7]. Marked kyphosis with a short neck and diffuse idiopathic skeletal hyperostosis are not described as risk factors, but they plausibly hindered axial alignment at intubation and later made cervical access complex. Once rupture has occurred, emergency intubation carries roughly a threefold increase in mortality [6]. The timing cannot be established. No emphysema was documented before transfer, it was extensive on arrival, and nothing was recorded during transport.

The lesion at operation was substantially larger than at bronchoscopy the previous day, and we do not base that claim on the recorded lengths. The 4 cm was a visual estimate made through a trachea occupied by a tube, and bronchoscopy cannot inspect the segment that the tube and cuff cover. The operative extent was never measured, and computed tomography mapped the air without delineating the tear. Two findings support extension independently of any estimate. Bronchoscopy recorded no injury below the tube at 22 cm at the lips, yet at thoracotomy, the laceration reached to within 1 cm of the carina, within the segment inspected the previous day. A cuff at 22 cm also abolished the leak on day 1, which is possible only if the whole defect lay proximal to it, an inference the intensivist recorded at the time; at operation, the lesion extended well distal to that depth and leaked. A tear spanning the cuff cannot be sealed at any depth, because a cuff cannot appose a torn wall. All of this preceded any operative manoeuvre, ending with the frank leak present on arrival in theatre. Rupture length independently predicts all-cause, though not rupture-related, mortality, with a reported threshold near 4.5 cm [15]; the operative lesion exceeded that threshold, and the bronchoscopic estimate fell below it by less than the margin of error of a visual estimate.

By the revised morphological classification, this was a Level II laceration, full thickness with subcutaneous or mediastinal emphysema, without the soft-tissue herniation that Level IIIA additionally requires, the oesophageal injury or mediastinitis that define Level IIIB, or the loss of substance that defines Level IV [10]. The original definitions offered no exact category, since Level II then stopped at the muscular wall and Level IIIA required herniation [9]; the revision's redefinition of Level II as full thickness closes that gap, and it is the classification we apply. Non-operative management spans Levels I to IIIA and was used for almost all such injuries in the validated series, with no mortality [10]. A systematic review found surgery associated with higher mortality where rupture was diagnosed outside theatre, a comparison open to confounding by indication [6]. None of the conditions that favour non-operative management [8,16] persisted here. Positive-pressure ventilation was unavoidable for an unresolved coma, the cuff seal was positional, and the emphysema, stable when repair was planned, was progressing by the morning of operation. Nor did she meet the criteria for endoscopic fibrin glue, which require a small superficial tear of the upper or middle trachea in a stable spontaneously breathing patient [17]; hers was full thickness, reached the carina, and she was ventilated. Stenting is described for patients judged poor surgical candidates rather than by lesion length [7].

The largest recent surgical series prefers cervical access where feasible, even for injuries reaching the main bronchi, with no procedure-related mortality, although only two thirds of patients left intensive care alive [18]. Here, thoracotomy was chosen, the cervical component could not be reached, and the leak from it was central to the fatal sequence. A cervical or combined approach merits consideration whenever a laceration is known preoperatively to reach the thoracic inlet. Intraoperative extracorporeal support is described where conventional ventilation cannot maintain oxygenation during airway surgery [19], and was used in a minority of that series [18]. Her reserve was already minimal before the chest was opened, with the right lung collapsed, a finding attributed radiologically to the hiatal hernia, and the left lung, the only one available for one-lung ventilation, compressed by an effusion. Extracorporeal support was not used, and in a patient for whom one-lung ventilation proved unsurvivable, it should have been considered before operation.

The terminal event carries the most generalisable lesson. The exchange was made over an airway exchange catheter under simultaneous videolaryngoscopy, with the glottis in view, yet the catheter passed extraluminally. Videolaryngoscopy shows the larynx, not the subglottic and cervical trachea, which is where the false passage occurred. With an unrepaired cervical laceration, glottic visualisation is false reassurance; only intraluminal confirmation that the catheter lies beyond the lesion, or avoidance of the exchange altogether, addresses the hazard.

This report has limitations. No Mallampati or Cormack-Lehane grade was recorded, the referring procedural details listed above were unavailable, and neither the exact time of intubation nor the transport duration was recorded. Respiratory mechanics and leak fraction were never measured. Predicted body weight was derived from the height recorded on the patient's identity document, which in marked kyphoscoliosis probably overstates stature, so every mL/kg value is a conservative underestimate; volumes are indexed to a predicted weight of 52.4 kg and vasopressor doses to an actual 89 kg. The operative extent was not measured, so progression is argued from the two findings above rather than from a change in length. Intervals between events are derived from the timing of clinical notes. The cause of the coma was never established, no lumbar puncture or post-mortem was performed, and influenza-associated encephalopathy, hypothermia, unwitnessed head injury from the two falls and beta-blocker toxicity were never excluded.

## Conclusions

Massive pneumomediastinum in a recently intubated patient should prompt urgent evaluation for central airway injury. The absence of pneumothorax does not exclude such injury, although it is not specific for tracheal rather than parenchymal disruption, since alveolar rupture with central air tracking can produce the same pattern. Computed tomography and bronchoscopy are complementary rather than sequential, and here neither established the true extent of the laceration.

A post-intubation tracheal laceration may extend substantially between initial endoscopic assessment and operation, and here, the course consistent with extension was documented before the chest was opened, so the extension cannot be attributed to the operation itself. The extent seen at first bronchoscopy should not be treated as fixed, and a patient who appears suitable for conservative management on initial findings may cease to be a candidate. Where the cuff seal is positional rather than secure, and positive-pressure ventilation cannot be discontinued, that possibility should be anticipated rather than discovered at operation, and the airway strategy for repair, including extracorporeal support where one-lung ventilation may not be tolerated, planned accordingly. Finally, when a cervical laceration remains unrepaired, exchange of the tracheal tube over an airway exchange catheter carries a risk of extraluminal passage that videolaryngoscopic control of the glottis does not mitigate, because the false passage occurs below the field of view.
